# Effect of transverse gap-junction channels on transverse propagation in an enlarged PSpice model of cardiac muscle

**DOI:** 10.1186/1742-4682-3-14

**Published:** 2006-03-16

**Authors:** Lakshminarayanan Ramasamy, Nicholas Sperelakis

**Affiliations:** 1 Dept. of Electrical Computer Engineering and Computer Science, University of Cincinnati College of Engineering, Cincinnati, OH 45219, USA; 2 Dept. of Molecular & Cellular Physiology, University of Cincinnati College of Medicine, Cincinnati, OH 45267-0576, USA

## Abstract

**Background:**

In previous PSpice modeling studies of simulated action potentials (APs) in parallel chains of cardiac muscle, it was found that transverse propagation could occur between adjacent chains in the absence of gap-junction (gj) channels, presumably by the electric field (EF) generated in the narrow interstitial space between the chains. Transverse propagation was sometimes erratic, the more distal chains firing out of order.

**Methods:**

In the present study, the propagation of complete APs was studied in a 2-dimensional network of 100 cardiac muscle cells (10 × 10 model). Various numbers of gj-channels (assumed to be 100 pS each) were inserted across the junctions between the longitudinal cells of each chain and between adjacent chains (only at the end cells of each chain). The shunt resistance produced by the gj-channels (R_gj_) was varied from 100,000 MΩ (0 gj-channels) to 1,000 MΩ (10 channels), 100 MΩ (100 channels) and 10 MΩ (1,000 channels). Total propagation time (TPT) was measured as the difference between the times when the AP rising phase of the first cell (cell # A1) and the last cell (in the J chain) crossed 0 mV. When there were no gj-channels, the excitation was transmitted between cells by the EF, i.e., the negative potential generated in the narrow junctional clefts (e.g., 100 Å) when the prejunctional membrane fired an AP. For the EF mechanism to work, the prejunctional membrane must fire a fraction of a millisecond before the adjacent surface membrane. When there were many gj-channels (e.g., 100 or 1,000), the excitation was transmitted by local-circuit current flow from one cell to the next through these channels.

**Results:**

TPT was measured as a function of four different numbers of transverse gj-channels, namely 0, 10, 100 and 1,000, and four different numbers of longitudinal gj-channels, namely 0, 10, 100 and 1,000. Thus, 16 different measurements were made. It was found that increasing the number of transverse channels had no effect on TPT when the number of longitudinal channels was low (i.e., 0 or 10). In contrast, when the number of longitudinal gj-channels was high (e.g., 100 or 1,000), then increasing the number of transverse channels decreased TPT markedly.

**Conclusion:**

Thus, complete APs could propagate along a network of 100 cardiac muscle cells even when no gj-channels were present between the cells. Insertion of transverse gj-channels greatly speeded propagation through the 10 × 10 network when there were also many longitudinal gj-channels.

## Introduction

Several different cardiac muscle preparations lack low-resistance connections between the cells [1,2]. Specifically, gap-junctions appear to be absent from lower vertebrates such as reptiles, amphibians and fish [2]. They also appear to be absent from some regions of the hearts of higher vertebrates and during embryonic development. When present, the gj-channels are mainly located between the cells in the longitudinal direction. However, transverse gj-channels have been described in a few cases [2].

In a computer simulation study of propagation in cardiac muscle, it was shown that the electric field (EF) that is generated in the narrow junctional clefts when the prejunctional membrane fires an action potential (AP) depolarizes the postjunctional membrane to its threshold [2-4]. Others have also proposed propagation by mechanisms that do not require low-resistance connections [5]. This results in excitation of the postjunctional cell after a brief junctional delay. The total propagation time (TPT) consists primarily of the summed junctional delays. This results in a staircase-shaped propagation, the surface sarcolemma of each cell firing almost simultaneously [4]. Propagation has been shown to be discontinuous (or saltatory) in cardiac muscle [6-9]. Fast Na^+ ^channels are localized in the junctional membranes of the intercalated disks [5,10,11], a requirement for the EF mechanism to work [1,3,4]. In connexin-43 and Cx40 knockout mice, propagation in the heart still occurs, but it is slowed [12-15] as predicted by our PSpice simulation studies [16]. Therefore, propagation is only slowed somewhat in the absence or paucity of gap junctions. Simulation of cardiac muscle APs using the PSpice program for circuit design and analysis showed that the EF developed in the junctional cleft is sufficiently large to allow excitation to be transferred without the requirement for a gap junction [16,17].

The purpose of the present study was to determine the effect of gap-junction channels on transverse propagation of complete action potentials (APs) through an enlarged network of cardiac muscle cells (10 × 10 model). The gj-channels were inserted between the longitudinal cells of each chain, and between the adjacent chains at certain points (namely at the two ends of each chain).

## Methods

**Figure 1:**
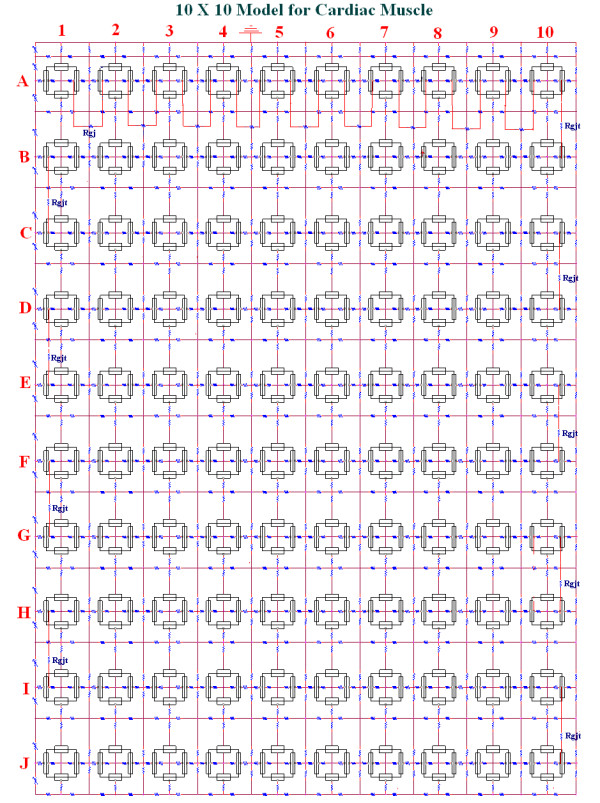
Block diagram of circuit used for the 10 × 10 model of 100 cardiac muscle cells bathed in Ringer solution. There were 10 parallel chains (A through J), each containing 10 cells (cells 1 through 10). Electrical stimulation (0.25 nA, 0.5 ms rectangular current pulses) was applied to the inside of the first cell of the first chain (cell #A1). The AP propagated from the stimulated cell #A1 through the entire network. A variable shunt resistance (Rgj) was inserted across each of the nine longitudinal cell junctions of each chain to reflect various numbers of gap-junction channels (0, 10, 100 and 1,000). This is depicted only for the A-chain (to enhance clarity of the figure). The radial resistance of the very narrow junctional cleft (Rjc) is depicted. Each cardiac cell is depicted by four basic units: two for the surface membrane (one upward-facing and one downward-facing) and one for each of the two end junctional membranes. This is more clearly illustrated in Figure 2. To reduce complexity, the transmembrane voltage (V_m_) was recorded from only the upward-facing surface membrane. The data illustrated in Figures 3 and 4 are records from only cells # 1, 5 and 10.

**Figure 2:**
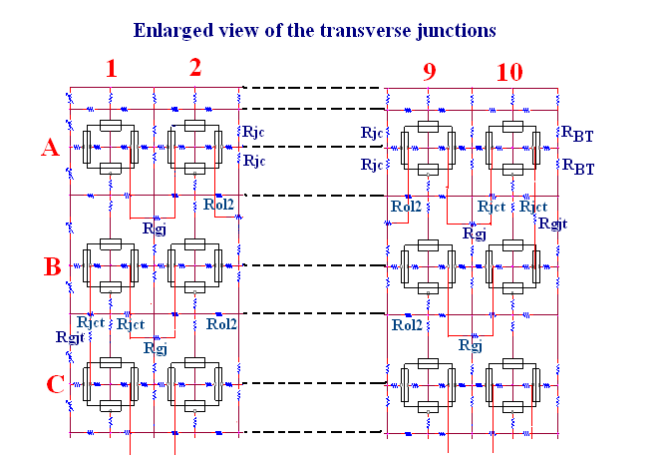
Enlarged view of the upper left and upper right portions of the complete circuit to illustrate the locations of the transverse gap-junctions. A total of 9 gap-junctions were positioned in a zigzag pattern across the 10 × 10 model. These were located between cells A10 and B10, B1 and C1, C10 and D10, D1 and E1, E10 and F10, F1 and G1, G10 and H10, H1 and I1, I10 and J10. In each transverse junction, R_jct _has a much higher value than R_ol2, _and it is equivalent to R_jc _in the longitudinal gap-junctions. R_gjt _is the shunt resistance for the transverse gap-junctions, and is equivalent to R_gj _for the longitudinal gap-junctions. Assuming a conductance of 100 pS for each gj-channel, Rgj and Rgjt were varied from 100,000 MΩ (0 channels) to 1,000 MΩ (10 channels), 100 MΩ (100 channels) and 10 MΩ (1000 channels). R_BT _is the bundle termination resistance at each end of the bundle, and has the standard value 1.0 KΩ. The standard values for R_jc _and R_jct _are 25 MΩ (50 MΩ ÷ 2 in parallel). The standard value for R_ol2 _is 200 KΩ.

The detailed methods and circuit parameters used for cardiac muscle were described previously [16-20]. Figure 1 shows the 10 × 10 model, consisting of 10 parallel chains (chains A through J), each containing 10 cells (cells 1 through 10). As shown in Figure 2, there were two surface membrane units in each cell (one facing upwards and one inverted) and one unit for each junctional membrane. The values of the circuit parameters used (standard conditions) are listed in Table 1 for both the surface and junctional units, and are consistent with those used previously [16,17,19,20]. The basic membrane units were interconnected by internal and external resistive networks. Thus, the seemingly complex overall circuit is, in reality, a series of repeat units. Additional details are given in our earlier papers [16].

**Table 1:** Parameter values used under standard conditions.

**Parameters**	**Surface unit**	**Junctional Unit**
C_m _(fF)	300	30
R_K _(MΩ)	71	710
R_Na _(MΩ)	710	7100
E_K _(mV)	-94	-94
E_Na _(mV)	+60	+60
R_d _(MΩ)	5000	5000
C_d _(pF)	30	30

	**Common**	

R_or _(KΩ)	1.0	
R_ol _(KΩ)	1.0	
Ri (KΩ)	100	
R_jc _(MΩ)	20 (40/2)	
R_BT _(KΩ)	1.0	

C_m _= Total cell capacitance

R_K _= Potassium resistance

R_Na _= Sodium resistance

E_K _= Potassium equilibrium potential

E_Na _= Sodium equilibrium potential

R_d _= Resistance in delay circuit

C_d _= Capacitance in delay circuit

R_or _= Radial resistance of external fluid

R_ol _= Longitudinal resistance of external fluid

R_i _= Longitudinal resistance of intracellular fluid

R_jc _= Radial resistance of junctional cleft

R_BT _= Bundle termination resistance

The cardiac muscle cell was assumed to be a cylinder 150 μm long and 16 μm in diameter. The cell capacitance was assumed to be 100 pF, and the input resistance to be 20 MΩ. A junctional tortuosity (interdigitation) factor of 4 was assumed for the cell junction [16]. The junctional cleft potential (Vjc) is produced across Rjc, the radial resistance of the narrow and tortuous junctional cleft. The junctional cleft contained two radial resistances (Rjc) of 50 MΩ, each in parallel. The 25 MΩ assigned to Rjc reflects the thickness of the junctional gap (end-to-end) and the tortuosity factor. The circuit used for each unit was kept as simple as possible, using only those ion channels that set the resting potential (RP) and predominate during the rising phase of the AP. The RP was -80 mV, and the overshoot potential was +30 mV (AP amplitude of 110 mV). Because the PSpice program does not have a V-dependent resistance to represent the increase in Na^+ ^conductance in cardiac muscle cells during depolarization and excitation, this function was simulated by a V-controlled current source ("black-box", BB) in each of the basic circuit units. The current output of the BB, at various membrane voltages, was calculated assuming a sigmoidal relationship between membrane voltage and resistance between -55 mV and -30 mV, to mimic physiological conditions.

The entire AP waveform was achieved by inserting a second BB into the Na^+ ^leg of the basic unit [18,20]. The first BB mimics Na^+ ^activation, and the second mimics deactivation of the Na^+^-channel conductance. The latter allowed repolarization to occur. BB-2 is connected between the outside and inside of the membrane unit, with reversed polarity compared to BB-1. The outputs of BB-1 and BB-2 were linked so that the output current of BB-2 nullified that of BB-1. BB-2 was activated with a delay time corresponding to the physiological delay value (i.e., to give an appropriate APD_50_). The required delay time was generated using a delay element R_d_C_d _(RC time constant). At the resting potential, the BB-2 output current was set to 0 nA. Once the cell has fired using BB-1's current, the potential across the input to BB-2 starts increasing with a rate corresponding to the RC time constant of the delay element. BB-2 then starts to respond to the rising voltage of the input. Two buffer elements (unity gain operational amplifiers) were added to isolate the input terminal of BB-2 from BB-1, to avoid interference between the two black boxes.

The network of 100 cells was assumed to be bathed in a large volume of Ringer solution connected to earth. The external resistance (R_o_) of this fluid consisted of two components: a radial resistance (R_or_) and a longitudinal resistance (R_ol_). The cells in the chain were either connected by low-resistance pathways (10, 100 or 1,000 gj channels) or not interconnected (0 channels), so that excitation could only be transmitted from one cell to the next by the EF developed in the junctional cleft. In some experiments, gj-channels were inserted in the transverse direction between the ends of adjacent chains. This allowed a zig-zag pattern of conduction through the network. If the transverse gj-channels were placed in the middle of each chain, then the conduction pattern would be in both directions from the transfer site, complicating the pattern analysis. Although gj-channels oriented in the transverse direction have not been extensively described, the dove-tailing of one myocardial cell with two contiguous longitudinal cells effectively gives a transverse spread of excitation.

The ends of the chains had a bundle termination resistance (R_BT_) of 1.0 KΩ to mimic physiological conditions. However, in some experiments, R_BT _was increased to 50 MΩ (to equal R_jc_: these data are not shown). This was done because, in experiments on single chains, there was a prominent edge-effect that was minimized by making R_BT _equal to R_jc _[21].

Electrical stimulation (rectangular current pulses of 0.25 nA and 0.50 ms duration) was applied to the inside of the first cell of the first chain of the network (cell #A1). To minimize confusion, the voltage was recorded from only one surface unit (upward-facing) in each cell, and from only 3 cells of each chain (cells #1, 5, and 10). TPT was measured as the difference between the times when the APs (rising phase) of the first cell and last cell crossed 0 mV. The PSpice program was set for a maximum step size for calculations of 100 μS. This is important because we found that the step size actually affected the results.

## Results

**Figure 3:**
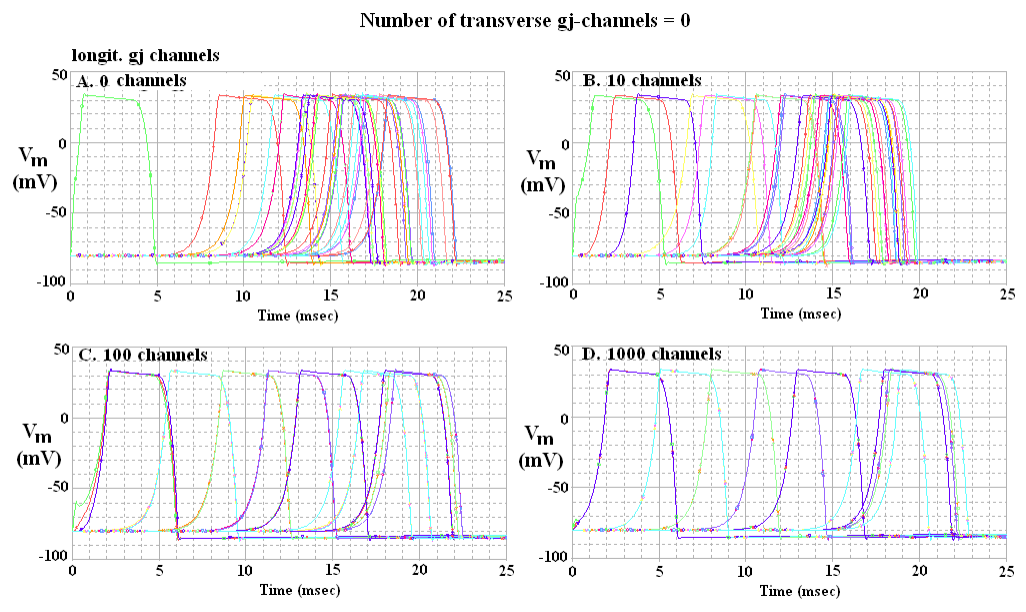
Propagation of APs simulated by PSpice through the 10 × 10 network of 100 cardiac muscle cells. There were zero transverse gj-channels (Rgj = 100,000 MΩ). The termination resistance at the ends of the chain (R_bt_) was 1.0 KΩ, similar to that for the fluid bathing the surface of the network. The 4 panels show the effect of varying the number of longitudinal gj-channels in each chain from zero (panel A, R_gj _= 100,000 MΩ) to 10 (panel B, R_gj _= 1,000 MΩ), 100 (panel C, R_gj _= 100 MΩ) and 1,000 (panel D, R_gj _= 10 MΩ). Note the presence of a hyperpolarizing after-potential following the repolarizing phase of the AP. When there were many gj-channels (C, D), the APs of all 10 cells in each chain were superimposed, indicating extremely fast longitudinal propagation within each chain. Therefore, only 10 traces are evident, one for each chain. Note that the TPT was slightly lengthened for 100 and 1,000 gj-channels compared to 10 channels (i.e., a higher degree of cell coupling actually inhibited the overall propagation velocity).

Figure 3 illustrates the AP waveform and propagation through the network of 100 cells with a strand termination resistance (R_bt_) of 1.0 KΩ. Propagation was studied with various numbers of gj-channels (0, 10, 100 and 1,000) traversing the junctions between the longitudinal cells of each chain. In addition, similar numbers of gj-channels were inserted transversely between the end cells of adjacent parallel chains, namely between cells A10 and B10, B1 and C1, C10 and D10, D1 and E1, E10 and F10, F1 and G1, G10 and H10, H1 and I1, I10 and J10. Assuming that each gj-channel has a conductance of 100 pS, these channels corresponded to a shunt resistance across each junction (R_gj_) of 100,000 MΩ (0 channels), 1,000 MΩ (10 channels), 100 MΩ (100 channels) and 10 MΩ (1,000 channels). The corresponding records are shown in panels A, B, C and D of Figure 3, respectively.

Figure 3 illustrates the records obtained when there were no transverse gj-channels (Rgjt = 100,000 MΩ). When there were no longitudinal gj-channels (Fig. 3A), or only 10 channels (Fig. 3B), fast propagation still occurred, mediated by the EF mechanism. When there are many longitudinal gj-channels (100 (C) or 1,000 (D)), the APs of all 10 cells in each chain are superimposed. Thus, there are only 10 traces in each panel. This figure clearly demonstrates that inserting many longitudinal junctions in the absence of transverse gj-channels had little effect on overall TPT. In fact, TPT was slightly increased when many longitudinal gj-channels were added.

**Figure 4:**
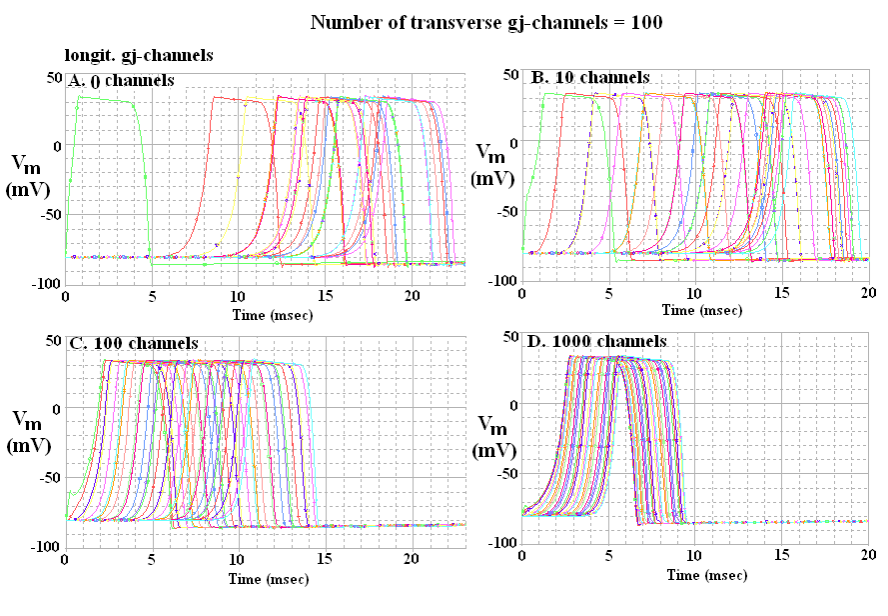
Propagation of cardiac APs simulated by PSpice through the 10 × 10 network when there were 100 transverse gj-channels in each of the nine transverse junctions. The transversely oriented gap junctions were located between the following cells: A10-B10, B1-C1, C10-D10, D1-E1, E10-F10, F1-G1, G10-H10, H1-I1 and I10-J10. The 4 panels illustrate the effect of varying the number of longitudinal gj-channels in each of the 10 parallel chains from 0 (panel A) to 10 (B), 100 (C) and 1000 (D). When there were many longitudinal gj-channels (e.g., 1,000, panel D), the APs of all 10 cells in each chain were superimposed, indicating that all 10 cells of each chain fired simultaneously. Hence, only 10 traces are evident. Thus, when the number of transverse gj-channels was substantial (e.g., 100), the overall TPT was markedly decreased when the number of longitudinal channels was increased.

Figure 4 illustrates the records obtained when there were many transverse gj-channels, namely 100. The APs of all 10 cells are superimposed in panel D, and nearly superimposed in panel C. This figure clearly demonstrates that TPT was markedly decreased when many longitudinal gj-channels were inserted, if many transverse gj-channels were also present.

**Figure 5:**
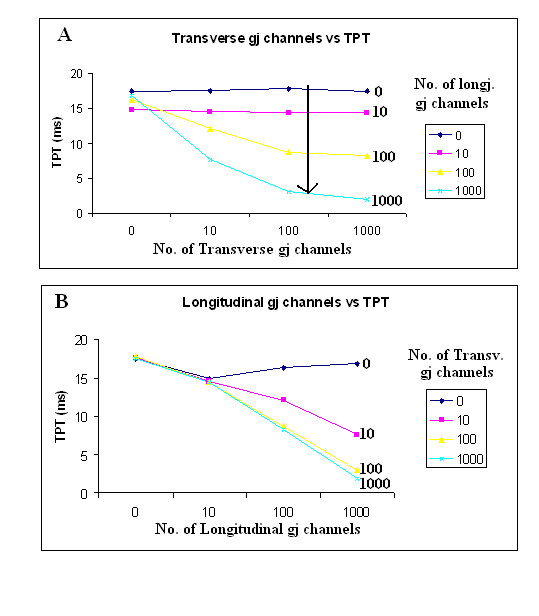
Graphic summary of the total propagation time (TPT) through the network of 100 cells as a function of the number of transverse gj-channels (A) or the number of longitudinal gj-channels (B). TPT is the difference between the times when the APs of cell #A1 and the last cell on the J chain crossed a V_m _of 0 mV. The shorter the TPT, the faster the propagation velocity. Assuming a gj-channel conductance of 100 pS, the R_gj _values of 10, 100, 1,000 and 100,000 MΩ correspond to 1,000, 100, 10 and 0 gj-channels, respectively. As in Figures 3 and 4, the graphic plots in panel A show that when there were no or few (0 or 10) longitudinal gj-channels, adding many transverse gj-channels had no effect on TPT. In contrast, when there were many longitudinal channels (100 or 1,000), adding many transverse channels markedly shortened the TPT. Panel B, which is a replot of the data in panel A, demonstrates that in the absence of transverse gj-channels, the TPT was actually slightly increased when there were many gj-channels (100 or 1,000).

Figure 5 shows graphs of TPT as a function of the number of transverse (A) and longitudinal (B) gj-channels. Panel A shows that increasing the number of transverse gj-channels decreases the TPT only when there are many longitudinal gj-channels (e.g., 100 or 1,000). Panel B is a replot of the data in panel A, and shows that increasing the number of longitudinal gj-channels decreases TPT only when there are numerous transverse gj-channels (e.g., 10, 100 or 1,000). Adding more and more longitudinal channels slightly lengthened TPT when there were no transverse channels. These data are also summarized in Table 2 to facilitate quantitative comparison.

**Table 2:** Summary of Total Propagation Time (TPT) and Velocity (θ) for Various combinations of longitudinal and transverse gap-junction channels in the 10 × 10 model for cardiac muscle.

**No. of channels**		**TPT**	^#^**Apparent Transv. velocity**	^##^**Overall velocity**	**Sequence of firing (Percent in order)**
	
**Longit.**	**Transv.**	**(ms)**	**(cm/sec)**	**(cm/sec)**	
0	0	17.5	0.82	84.9	23
0	10	17.6	0.82	84.4	13
0	100	17.8	0.81	83.4	13
0	1000	17.5	0.82	84.9	13
					
10	0	14.8	0.97	100	47
10	10	14.5	0.99	102	60
10	100	14.4	1.00	103	63
10	1000	14.4	1.00	103	63
					
100	0	16.3	0.88	91.1	100
100	10	12.1	1.19	123	100
100	100	8.7	1.66	171	100
100	1000	8.2	1.76	181	100
					
1000	0	16.8	0.86	88.4	100
1000	10	7.6	1.89	195	100
1000	100	3.0	4.80	495	100
1000	1000	1.9	7.58	782	100

^# ^Apparent transverse velocity was calculated from the TPT assuming a cell diameter of 16 μm.

Velocity (θ) = (16 μm/junction) × (9 junctions transversely)/TPT

^## ^Overall velocity was calculated from the TPT assuming a cell length of 150 μm.

Velocity (θ) = (150 μm/junction) × (99 junctions)/TPT

**Figure 6:**
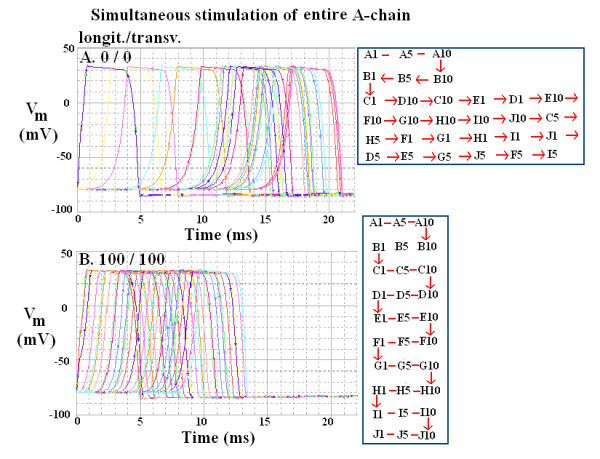
Transverse propagation of simulated cardiac APs in the 10 × 10 network when the entire A-chain was stimulated simultaneously. Only two examples are depicted out of the total of 16 combinations that were run. A: The ratio of longitudinal to transverse gj-channels was 0/0, B: The ratio of longitudinal to transverse gj-channels was 100/100. As in Figures 3 and 4, the records from only 3 cells (cells #1, 5, and 10) in each chain are shown. The sequence of firing of the 15 cells selected is given in the inset table in each panel. When the sequence of the cells is in order there is a zigzag pattern, because the transverse gj-channels were inserted only at the ends of each chain.

In order to measure the apparent transverse velocity more precisely, all 10 cells of the entire A-chain were stimulated simultaneously. This procedure eliminated the time required for propagation within the A-chain (which occurs when only cell A1 is stimulated). The results were very similar to those found when only cell A1 was stimulated. Therefore, only two of the 16 combinations are illustrated in Figure 6. Panel A shows the records obtained when there were no gj-channels, either longitudinal or transverse. The TPT was 16.5 ms, as compared to 17.5 in Figure 3A. Thus, stimulating the entire A-chain reduced the TPT by 1.0 ms, by eliminating the time required for propagation within the A-chain. Panel B of Figure 6 shows the records obtained when there were 100 gj-channels in both the longitudinal and the transverse direction. The TPT was 8.9 ms, as compared to 8.5 ms in Figure 4C. Thus, stimulating the entire A-chain actually increased the TPT slightly (by 0.4 ms). This was due to an increase in the delay time for firing of the first trace, presumably because when all 100 cells are fairly well coupled resistively, the increased capacitance makes the stimulating current less effective owing to the prolonged RC time constant.

## Discussion

The present study reveals several new facets of the factors influencing the velocity of transverse propagation between parallel strands of cardiac muscle cells. First, the PSpice model has been expanded to a 10 × 10 network of 100 cells, as compared to the previous 7 × 7 model of 49 cells; so the number of cells has been doubled and the model includes longer chains and more parallel chains. This should reduce the edge (boundary) effects and provide greater accuracy. However, this enlarged 10 × 10 model is still far short of mimicking the physiological condition.

Second, the APs are now complete, with repolarization instituted in addition to depolarization. Thus, the simulated APs have both a depolarizing and a repolarizing phase. Hence, the transverse propagation of repolarization can now be studied.

Third, gj-channels have been inserted in the transverse direction for the first time. Previously, gj-channels were inserted only in the longitudinal direction, i.e., between the myocardial cells lying end-to-end within each chain. The transverse gap junctions were positioned at the ends of each chain, so that propagation occurred in a zigzag pattern. Thus, the end chains A and J had only one gap junction each, whereas chains B-I had two transverse junctions, one at each end. The results were quantitated by varying the number of transverse gj-channels (namely 0, 10, 100 and 1,000) while the number of longitudinal gj-channels was held constant (at 0, 10, 100 and 1,000). Thus, there were 16 different combinations. These are plotted in Figure 5, as a function of the four different numbers of transverse gj-channels (panel A) or the four different numbers of longitudinal gj-channels (panel B).

Panel A of Figure 5 shows that the presence of many (or few) transverse channels had no effect on TPT (and hence on transverse propagation velocity) when there were no or only few (i.e., 0 or 10) longitudinal channels. However, when there were many (100 or 1,000) longitudinal channels, the transverse channels had a marked effect on TPT. At a fixed number of transverse channels (10, 100 or 1,000), adding longitudinal channels decreased TPT more and more (vertical arrow in panel A).

Panel B of Figure 5 replots the data in panel A to show TPT as a function of the four different numbers of longitudinal channels, with the number of transverse channels held constant at each of the four levels. Plotting the data in this way clearly shows that when the number of transverse channels was 10, 100, or 1,000, increasing the number of longitudinal channels decreased TPT. However, when there were no transverse channels, inserting more and more longitudinal channels had no effect. In fact, there was actually a small increase in TPT as more longitudinal channels were inserted. This is in agreement with our previous report [17]. We explain this finding as follows. When there is strong longitudinal coupling, the transverse transfer energy must be greater because the entire chain of 10 cells must be brought to threshold simultaneously. In contrast, when longitudinal coupling is weak, then if the transfer energy were sufficient to activate only one cell in the chain, this activated cell would, in turn, spread excitation to the other cells of the chain.

In summary, we have determined the effect of inserting transverse gj-channels on transverse propagation velocity, using an expanded model (10 × 10) of cardiac muscle with complete APs (repolarizing as well as depolarizing phases). When there were no transverse gj-channels, inserting many longitudinal gj-channels had a negligible effect on TPT and overall propagation velocity. In fact, there was a small increase in TPT when more and more longitudinal channels were inserted. In contrast, when there were many transverse channels (e.g., 100), inserting more and more longitudinal channels greatly decreased TPT. Hence, the effect of transverse channels was variable, depending on the number of longitudinal channels present.

## References

[B1] Sperelakis N (2002). An electric field mechanism for transmission of excitation between myocardial cells. Circ Res.

[B2] Sperelakis N, McConnell K (2002). Electric field interactions between closely abutting excitable cells. IEEE-Eng Med Biol.

[B3] Sperelakis N, Mann JE (1977). Evaluation of electric field changes in the cleft between excitable cells. J Theor Biol.

[B4] Picone JB, Sperelakis N, Mann JE (1991). Expanded model of the electric field: Hypothesis for propagation in cardiac muscle. Math and Computer Modeling.

[B5] Kucera JP, Rohr S, Rudy Y (2002). Localization of sodium channels in intercalated disks modulates cardiac conduction. CircRes.

[B6] Spach MS, Miller WT, Geselowitz DB, Barr R, Kootsey JM, Johnson EA (1981). The discontinuous nature of propagation in normal canine cardiac muscle. Evidence for recurrent discontinuity of intracellular resistance that affects the membrane currents. Circ Res.

[B7] Diaz PJ, Rudy Y, Plonsey R (1983). Intercalated discs as a cause for discontinuous propagation in cardiac muscle. A theoretical simulation. Ann Biomed Eng.

[B8] Shaw RM, Rudy Y (1997). Ionic mechanisms of propagation in cardiac tissue. Roles of the sodium and L-type calcium currents during reduced excitability and decreased gap junction coupling. Circ Res.

[B9] Henriquez AP, Vogel R, Muller-Borer BJ, Henriquez CS, Weingart R, Cascio WE (2001). Influence of dynamic gap junction resistance on impulse propagation in ventricular myocardium: a computer simulation study. Biophys J.

[B10] Cohen SA (1994). Immunocytochemical localization of rH1 sodium channel in adult rat heart atria and ventricle. Presence in terminal intercalated disks. Circulation.

[B11] Sperelakis N (1995). Cable properties and propagation of action potentials. CH 18. Cell Physiology Source Book.

[B12] Morley GE, Vaidya D, Samie FH, Lo CW, Taffet SM, Delmar M, Jalife J (1999). Characterization of conduction in the ventricles of normal and heterozygous Cx43 knockout mice using optical mapping. J Cardiovasc Electrophysiol.

[B13] Tamaddon HS, Vaidya D, Simon AM, Paul DL, Jalife J, Morley GE (2000). High resolution optical mapping of the right bundle branch in connexin40 knockout mice reveals low conduction in the specialized conduction system. Circ Res.

[B14] Gutstein DE, Morley GE, Tamaddon H, Vaidya D, Schneider MD, Chen J, Chien KR, Stuhlmann H, Fishman GI (2001). Conduction slowing and sudden arrythmic death in mice with cardiac restricted inactivation of connexin43. Circ Res.

[B15] Vaidya D, Tamaddon HS, Lo CW, Taffet SM, Delmar M, Morley GE (2001). Null mutation of connexin43 causes slow propagation of ventricular activation in the late stages of mouse embryonic development. Circ Res.

[B16] Sperelakis N, Ramasamy L (2002). Propagation in cardiac muscle and smooth muscle based on electric field transmission at cell junctions: An analysis by PSpice. IEEE-Eng Med Biol.

[B17] Sperelakis N, Murali KPV (2003). Combined electric field and gap junctions on propagation of action potentials in cardiac muscle and smooth muscle in PSpice simulation. J Electrocardiol.

[B18] Sperelakis N, Ramasamy L, Kalloor B (2005). Propagated repolarization of simulated action potentials in cardiac muscle and smooth muscle. Theor Biol Med Modeling.

[B19] Ramasamy L, Sperelakis N Action potential repolarization enabled by Ca^++ ^deactivation in PSpice simulation of smooth muscle propagation. Bio Med Eng Online.

[B20] Ramasamy L, Sperelakis N (2005). Repolarization of the action potential enabled by Na+ channel deactivation in PSpice simulation of cardiac muscle propagation. Theor Biol Med Modeling.

[B21] Sperelakis N, Kalloor B, Ramasamy L (2005). Boundary effects influence velocity of transverse propagation of simulated cardiac action potentials. Theor Biol Med Modeling.

